# Survival Prediction in Middle‐Aged and Elderly Patients With Burkitt Lymphoma: A Comprehensive Nomogram Approach Based on SEER Data

**DOI:** 10.1002/cam4.71334

**Published:** 2025-11-10

**Authors:** Xia Cao, Duanzong Zhang, Jichang Gong, Xin Yang, Jiqiong He, Yaqiong Li, Hongjiang Pu

**Affiliations:** ^1^ Department of Hematology Dazhou Central Hospital Dazhou Sichuan China; ^2^ The Third Affiliated Hospital of Kunming Medical University Peking University Cancer Hospital Yunnan Hospital, Yunnan Cancer Hospital Kunming China; ^3^ Department of Oncology Dazhou Central Hospital Dazhou Sichuan China

**Keywords:** Burkitt lymphoma, elderly, nomogram, prognostic model, SEER

## Abstract

**Background:**

Burkitt lymphoma (BL) in middle‐aged and elderly populations presents unique prognostic challenges due to distinct biological behaviors and therapeutic vulnerabilities. Current prognostic tools inadequately address age‐specific survival determinants in this understudied cohort.

**Methods:**

This study used the Surveillance, Epidemiology, and End Results (SEER) data from patients diagnosed with BL between 2000 and 2020, aged 45 years and older. Through univariate and multivariate Cox regression analyses, we identified independent prognostic factors affecting overall survival (OS) and cancer‐specific survival (CSS). Finally, nomograms were constructed, and the models were evaluated across three dimensions.

**Results:**

Multivariate analysis results indicated that factors such as age, race, Ann Arbor stage, and chemotherapy were independently associated with OS, while age, Ann Arbor stage, radiotherapy, chemotherapy, and number of tumor masses were independently associated with CSS. The nomogram model effectively predicted the 1‐, 3‐, and 5‐year probabilities of OS and CSS. The results from receiver operating characteristic curves, calibration curves, and decision curve analysis in the training and validation groups confirmed that the risk prediction nomogram could accurately predict the survival of BL patients.

**Conclusion:**

The nomogram model constructed in this study provides a personalized survival prediction tool for BL patients, effectively distinguishing the survival probabilities of different risk groups. This research offers new insights for risk stratification and treatment management of middle‐aged and elderly BL patients.

## 1 Introduction

Burkitt lymphoma (BL) is a highly aggressive non‐Hodgkin lymphoma (NHL), for which survival benefits rely on early detection, aggressive supportive care, and risk‐stratified multi‐agent chemotherapy [1]. Recent epidemiological studies from the Global Burden of Disease (GBD) indicate that by 2021, BL had high incidence and prevalence rates among children aged 0–14. However, the distribution of incidence and prevalence has also expanded to include middle‐aged and older populations [2]. In these older cohorts, BL primarily presents as sporadic or immunodeficiency‐associated subtypes, often affecting the gastrointestinal tract, bone marrow, and central nervous system (CNS) [3]. Additionally, middle‐aged and elderly patients often present with multiple comorbidities, compromised treatment tolerance, and distinct tumor biology, leading to ongoing controversies regarding optimal therapeutic strategies. Despite significant improvements in clinical cure rates with modern immunochemotherapy, the prognosis for older patients remains suboptimal [4, 5, 6], posing unique challenges for clinical management and prognostic evaluation.

The Ann Arbor staging system remains widely used for risk stratification in adult BL; however, it primarily stages disease based on tumor distribution and nodal involvement, without accounting for patient‐specific factors [7]. The recently proposed BL International Prognostic Index (BL‐IPI) incorporates clinical parameters—age, performance status, serum lactate dehydrogenase levels, and CNS involvement—to generate risk scores. However, its predictive validity derived from standard treatment protocols and demonstrated limited informative value for patients over 65 years old, thereby constraining its clinical utility in middle‐aged and elderly populations [8].

Nomograms have become an efficient and intuitive tool for predicting disease prognosis. By incorporating significant prognostic variables, these models enable visual assessment of patient outcomes while providing accurate calculations of individualized survival probabilities. Such predictive tools have gained widespread application in oncology outcome evaluation [9, 10, 11]. This study aimed to develop a comprehensive nomogram utilizing the BL dataset from the Surveillance, Epidemiology, and End Results (SEER) database, with particular emphasis on middle‐aged and elderly populations. The proposed model sought to equip clinicians with an enhanced predictive instrument for optimizing treatment strategies and improving outcome prediction accuracy in this specific patient demographic.

## 2 Materials and Methods

### 2.1 Data Source and Patient Enrollment

This retrospective cohort study employed data from the SEER, 22 registries research database, representing 34.9% of the US population. Patients aged ≥ 45 years with primary BL diagnosed between 2000 and 2020 were included, with follow‐up through January 1, 2021, to ensure ≥ 5‐year survival assessment. Cases of BL were identified using the histology code 9686 according to the International Classification of Diseases for Oncology, 3rd Edition (ICD‐O‐3).

**FIGURE 1:**
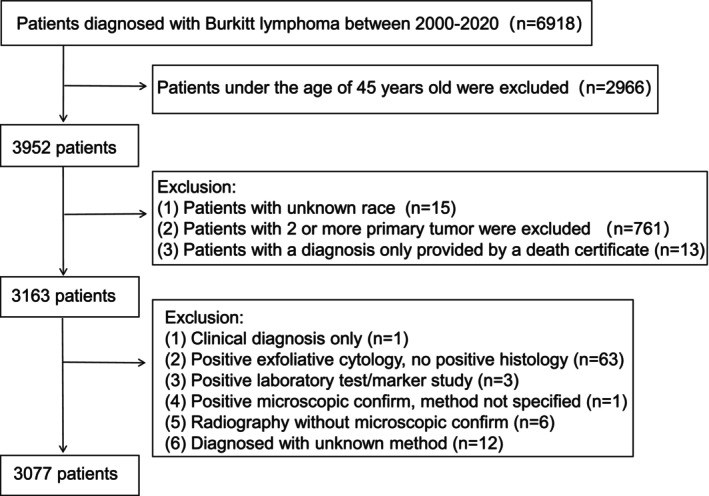
Sample selection process flowchart.

Inclusion criteria mandated complete demographic (age, race), clinical (Ann Arbor stage, treatment status), and outcome data, alongside confirmed histopathological diagnosis per institutional guidelines. Exclusion criteria encompassed: (1) incomplete clinical/outcome data, (2) concurrent diagnosis of other malignancies, (3) participation in experimental therapies, (4) prior lymphoma history, and (5) indeterminate diagnostic/treatment records. The final analytic cohort comprised 3077 BL patients (Figure 1).

### 2.2 Data Collection

Demographic variables included gender, age, race, marital status, and household income. Clinical variables comprised Ann Arbor stage, treatment modalities (radiation therapy, chemotherapy), tumor characteristics (primary site, laterality, number of tumor masses), diagnostic‐to‐treatment interval, and first primary malignancy status. Outcomes encompassed overall survival (OS) and cancer‐specific survival (CSS).

### 2.3 Nomogram Development and Analyses

The SEER cohort was randomly partitioned into training (70%) and validation (30%) sets. Univariate Cox regression identified 13 potential prognostic factors for OS and CSS. Variables with *p* < 0.05 underwent multivariate analysis via backward stepwise selection. Significant predictors were integrated into a nomogram using R (v4.0.2; “rms,” “survival” packages), assigning weighted scores to estimate 1‐, 3‐, and 5‐year survival probabilities.

Model discrimination was evaluated via time‐dependent ROC curves (AUC > 0.7 indicating strong performance). Calibration curves assessed nomogram‐predicted versus observed outcomes, while decision curve analysis (DCA) quantified clinical net benefit. Optimal risk stratification thresholds were determined using the *surv_cutpoint* method (Kaplan–Meier/log‐rank), classifying patients into low‐, intermediate‐, and high‐risk groups. Statistical analyses were performed in SPSS (v24.0; Cox regression) and R (v4.0.2; nomogram construction, ROC/DCA, survival curves). Two‐tailed *p* < 0.05 defined significance.

## 3 Results

### 3.1 Baseline Characteristics of Patients

The training (*N* = 2154) and validation (*N* = 923) cohorts demonstrated balanced baseline characteristics across 12 demographic/clinical variables (sex, age, race, marital status, Ann Arbor stage, etc.) (Table 1), with concordant distributions in: male predominance (69%), median age 62 years (IQR 52–74), White ethnicity (82%), marital status (57%), advanced‐stage disease (Ann Arbor IV: 55%), chemotherapy use (80%), and ≤ 1 month treatment initiation (88%). Nodal involvement (68%), non‐paired organ spread (91%), and solitary masses (25%) showed comparable prevalence. Significant between‐group differences emerged in household income distribution (mid range $50K–75K: 42.8% vs. 36.2%, *p* = 0.002) and radiotherapy utilization (10.0% vs. 7.8%, *p* = 0.048) (Table 1).

**TABLE 1:** Characteristics of BL patients included in the study and analysis of differences.

Characteristic	Cohort		*p*
	Training cohort	Validation cohort	
	*N* = 2154	*N* = 923	
Sex			0.796
Male	1499 (69.6%)	638 (69.1%)	
Female	655 (30.4%)	285 (30.9%)	
Age			0.536
Median (IQR)	62 (52, 73)	62 (53, 74)	
Race			0.844
White	1768 (82.5%)	766 (83.4%)	
Black	158 (7.4%)	53 (5.8%)	
Other^a^	218 (10.2%)	89 (9.7%)	
Marital status			0.599
Divorced	166 (8.0%)	80 (9.1%)	
Married	1189 (57.2%)	493 (55.9%)	
Separated	25 (1.2%)	16 (1.8%)	
Unmarried	484 (23.3%)	203 (23.0%)	
Widowed	214 (10.3%)	90 (10.2%)	
Household income			0.002
50K–75K	779 (36.2%)	395 (42.8%)	
Greater than 75K	1264 (58.7%)	486 (52.7%)	
Less than 50,000	111 (5.2%)	42 (4.6%)	
Ann Arbor stage			0.877
I	279 (18.2%)	127 (19.3%)	
II	237 (15.4%)	96 (14.6%)	
III	170 (11.1%)	69 (10.5%)	
IV	850 (55.3%)	366 (55.6%)	
Radiation			0.048
No	1986 (92.2%)	831 (90.0%)	
Yes	168 (7.8%)	92 (10.0%)	
Chemotherapy			0.245
No	418 (19.4%)	196 (21.2%)	
Yes	1736 (80.6%)	727 (78.8%)	
Diagnosis to treatment time			0.502
≤ 1 month	1506 (88.1%)	652 (89.1%)	
> 1 month	203 (11.9%)	80 (10.9%)	
Primary site			0.950
Lymphatic node	1462 (68.4%)	626 (68.3%)	
Extra‐lymph node	676 (31.6%)	291 (31.7%)	
Laterality			0.783
Unilateral	1960 (91.0%)	837 (90.7%)	
Bilateral	194 (9.0%)	86 (9.3%)	
First malignant primary			0.300
No	323 (15.0%)	152 (16.5%)	
Yes	1831 (85.0%)	771 (83.5%)	
Number of tumor masses			0.093
≥ 2	1657 (76.9%)	684 (74.1%)	
1	497 (23.1%)	239 (25.9%)	
CCS			0.201
Yes	1116 (51.8%)	455 (49.3%)	
No	1038 (48.2%)	468 (50.7%)	

^a^ Other refer to Asian/Pacific Islander or American Indian.

### 3.2 Nomogram Variable Screening

In univariate analysis for OS, age (HR = 1.04, 95% CI: 1.03–1.04, *p* < 0.001), White race (HR = 0.78, 95% CI: 0.64–0.96, *p* = 0.016), widowed marital status (HR = 2.12, 95% CI: 1.66–2.71, *p* < 0.001), household income < $50K (HR = 1.34, 95% CI: 1.06–1.70, *p* = 0.014), advanced Ann Arbor stage (stage III: HR = 1.52; stage IV: HR = 1.70; both *p* < 0.001), chemotherapy (HR = 0.24, 95% CI: 0.21–0.27, *p* < 0.001), bilateral laterality (HR = 0.70, 95% CI: 0.58–0.86, *p* < 0.001), and first primary malignancy (HR = 0.67, 95% CI: 0.59–0.77, *p* < 0.001) showed significant associations (Table S1). Multivariate analysis confirmed independent associations for OS with age (HR = 1.03, 95% CI: 1.03–1.04, *p* < 0.001), White race (HR = 0.74, 95% CI: 0.59–0.93, *p* = 0.009), progressive Ann Arbor stage (stage III: HR = 1.62; stage IV: HR = 1.85; all *p* ≤ 0.001), and chemotherapy (HR = 0.24, 95% CI: 0.20–0.28, *p* < 0.001) (Table 2).

**TABLE 2:** Multivariate COX regression analysis of OS and CSS predictors in training set.

Variables	OS			Variables	CSS		
	HR	95% CI	*p*		HR	95% CI	*p*
Age	1.03	1.03, 1.04	< 0.001	Age	1.01	1.01, 1.02	< 0.001
Race				Ann Arbor stage			
Black	—	—		I	—	—	
Other	0.95	0.71, 1.27	0.718	II	1.37	1.05, 1.79	0.022
White	0.74	0.59, 0.93	0.009	III	1.26	0.96, 1.65	0.095
Ann Arbor stage				IV	1.90	1.54, 2.34	< 0.001
I	—	—		Radiation			
II	1.18	0.94, 1.46	0.147	No	—	—	
III	1.62	1.28, 2.05	< 0.001	Yes	0.74	0.58, 0.94	0.013
IV	1.85	1.56, 2.20	< 0.001	Chemotherapy			
Chemotherapy				No	—	—	
No	—	—		Yes	0.31	0.26, 0.36	< 0.001
Yes	0.24	0.20, 0.28	< 0.001	Laterality			
Marital status				Unilateral	—	—	
Divorced	—	—		Bilateral	0.77	0.56, 1.05	0.101
Married	1.02	0.80, 1.29	0.899	Number of tumor masses			
Separated	1.88	1.11, 3.19	0.019	1	—	—	
Unmarried	1.10	0.85, 1.43	0.478	≥ 2	1.60	1.35, 1.91	< 0.001
Widowed	0.88	0.66, 1.18	0.401				
Household income							
50K–75K	—	—					
Greater than 75K	0.97	0.86, 1.11	0.683				
Less than 50,000	1.06	0.80, 1.40	0.684				
Laterality							
Unilateral	—	—					
Bilateral	0.84	0.67, 1.06	0.142				
First malignant primary							
No	—	—					
Yes	1.02	0.87, 1.20	0.820				

Abbreviations: CI, confidence interval; HR, hazard ratio.

For CSS, univariate analysis in the training cohort identified age (HR = 1.01, 95% CI: 1.01–1.02, < 0.001), stage IV Ann Arbor classification (HR = 1.54, 95% CI: 1.26–1.89, *p* < 0.001), radiation therapy (HR = 0.69, 95% CI: 0.55–0.86, *p* < 0.001), chemotherapy (HR = 0.34, 95% CI: 0.30–0.39, *p* < 0.001), bilateral laterality (HR = 0.64, 95% CI: 0.49–0.82, *p* < 0.001), and ≥ 2 tumor masses (HR = 1.42, 95% CI: 1.24–1.64, *p* < 0.001) as significant predictors (Table S2). Multivariate CSS analysis retained age (HR = 1.01, 95% CI: 1.01–1.02, *p* < 0.001), Ann Arbor stage II (HR = 1.37, *p* = 0.022) and IV (HR = 1.90, *p* < 0.001), radiation therapy (HR = 0.74, *p* = 0.013), chemotherapy (HR = 0.31, *p* < 0.001), and ≥ 2 tumor masses (HR = 1.60, *p* < 0.001) as independent prognostic factors (Table 2). Gender, race, marital status, income, treatment delay, primary site, and first primary malignancy status showed no significant CSS associations.

### 3.3 Nomogram Construction and Validation

**FIGURE 2:**
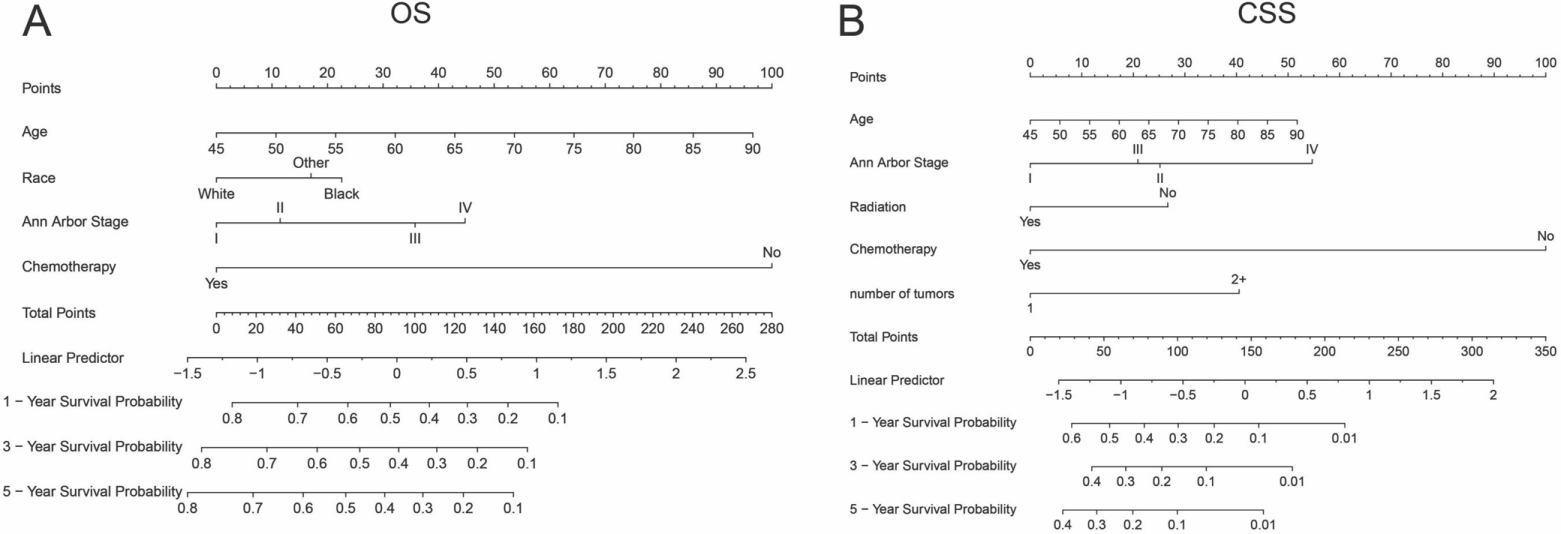
Prognostic nomograms to predict the 1‐, 3‐, and 5‐year overall survival (OS) (A) and cancer‐specific survival (CSS) (B) in middle‐aged and elderly BL patients.

Based on multivariate analysis results, predictive nomograms were constructed to estimate 1‐, 3‐, and 5‐year OS and CSS probabilities (Figure 2A,B). The OS nomogram (Figure 2A) incorporated age, race, Ann Arbor stage, and chemotherapy, with total scores derived from summed component points converted to survival probabilities via linear predictors. Similarly, the CSS nomogram (Figure 2B) integrated age, Ann Arbor stage, radiotherapy, chemotherapy, and tumor multiplicity (≥ 2 masses) to generate prognostic estimates.

**FIGURE 3:**
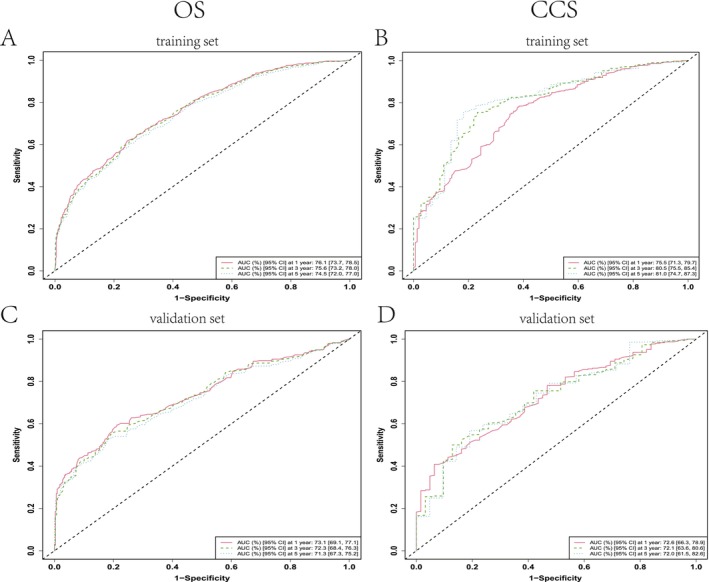
Receiver operating characteristic (ROC) curve analysis evaluating the nomogram's predictive performance for 1‐, 3‐, and 5‐year overall survival (OS) (A, C) and cancer‐specific survival (CSS) (B, D) in middle‐aged and elderly BL patients across training (A, B) and validation (C, D) cohorts.

Model discrimination was assessed using time‐dependent ROC analysis. In the training cohort, the OS model achieved AUCs of 0.761 (95% CI: 0.737–0.785), 0.756 (0.732–0.780), and 0.745 (0.720–0.770) for 1‐, 3‐, and 5‐year predictions, respectively (Figure 3A). Corresponding CSS AUCs were 0.755 (0.713–0.797), 0.805 (0.755–0.854), and 0.810 (0.747–0.873) (Figure 3B). Validation cohort performance remained robust, with OS AUCs of 0.731 (0.691–0.771), 0.723 (0.684–0.763), and 0.713 (0.673–0.752), and CSS AUCs of 0.726 (0.683–0.789), 0.721 (0.636–0.806), and 0.720 (0.615–0.826) at 1, 3, and 5 years (Figure 3C,D).

**FIGURE 4:**
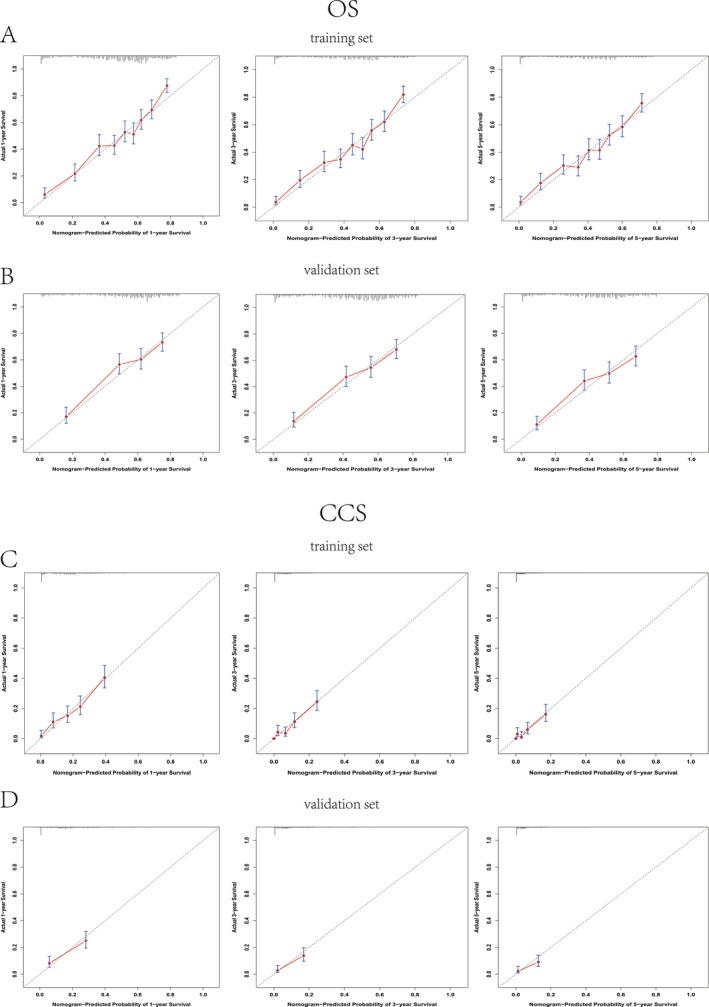
Calibration plots comparing predicted versus observed 1‐, 3‐, and 5‐year overall survival (OS) (A, B) and cancer‐specific survival (CSS) (C, D) probabilities in middle‐aged and elderly BL patients across training (A, C) and validation (B, D) cohorts.

Calibration curves demonstrated strong concordance between predicted and observed outcomes, with minimal deviation from the ideal 45° line across all time points for both OS and CSS in training and validation cohorts (Figure 4).

**FIGURE 5:**
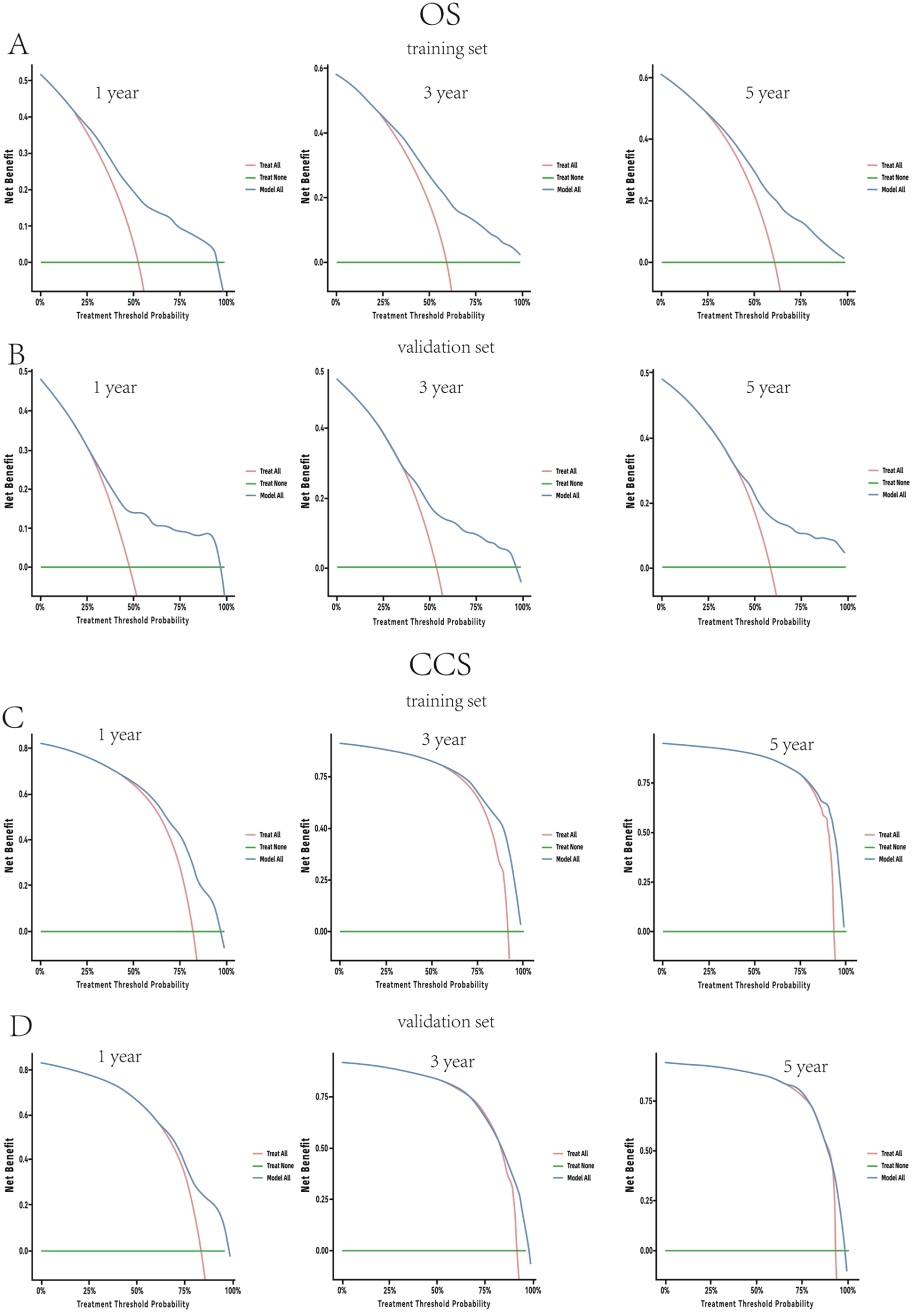
Decision curve analysis (DCA) evaluating the nomogram's clinical utility for 1‐, 3‐, and 5‐year overall survival (OS) (A, B) and cancer‐specific survival (CSS) (C, D) predictions in middle‐aged and elderly BL patients across training (A, C) and validation (B, D) cohorts.

DCA plots further confirmed clinical utility, as the nomograms provided superior net benefit over “treat‐all” or “treat‐none” strategies across threshold probabilities of 10%–80% (Figure 5).

### 3.4 Performance of the Nomogram in Stratifying Risk

Patients were classified into low‐, intermediate‐, and high‐risk groups using NomoScore tertiles. For OS, thresholds of 57.28 and 92.19 defined low‐risk (< 57.28), intermediate‐risk (57.28–92.19), and high‐risk (> 92.19) categories. CSS stratification employed thresholds of 124.87 and 155.32, yielding analogous risk groups.

**FIGURE 6:**
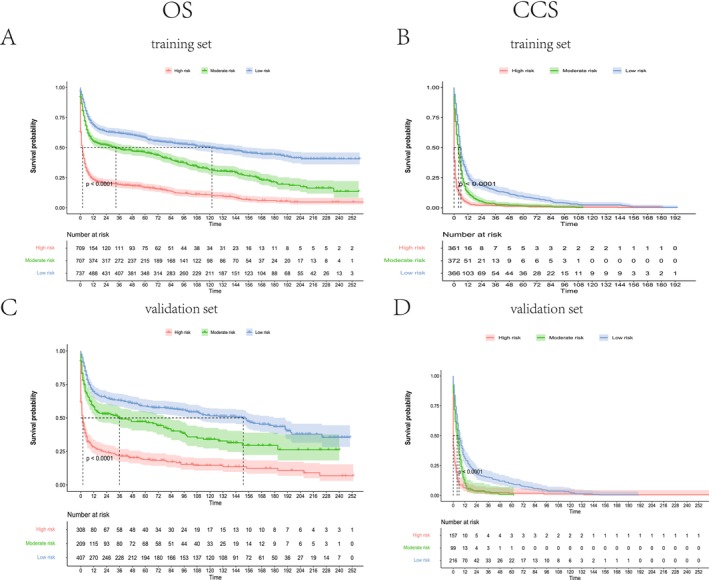
Kaplan–Meier survival curves for overall survival (OS) (A, C) and cancer‐specific survival (CSS) (B, D) in middle‐aged and elderly BL patients stratified into low‐, intermediate‐, and high‐risk groups, analyzed in the training (A, B) and validation (C, D) cohorts.

Kaplan–Meier analysis demonstrated significant survival disparities across risk strata in both training and validation cohorts (*p* < 0.0001). High‐risk patients exhibited steep survival declines, with 1‐, 3‐, and 5‐year OS probabilities of 0.20–0.25, 0.10–0.15, and 0.05–0.10, respectively, and CSS probabilities of 0.15–0.20, 0.08–0.10, and 0.03–0.05. Intermediate‐risk groups displayed moderate declines (OS: 0.40–0.45, 0.30–0.35, 0.25–0.30; CSS: 0.35–0.40, 0.25–0.30, 0.20–0.25 at 1, 3, and 5 years). Low‐risk patients maintained stable outcomes (OS: 0.60–0.65, 0.50–0.55, 0.45–0.50; CSS: 0.55–0.60, 0.50–0.55, 0.45–0.50) (Figure 6). These results validate the nomogram's capacity to discriminate clinically distinct prognostic subgroups, supporting its utility in risk‐adapted clinical decision‐making.

## 4 Discussion

BL in middle‐aged and elderly patients presents distinct clinical and prognostic challenges compared to younger cohorts, characterized by advanced‐stage disease prevalence (66% in our cohort), elevated treatment‐related toxicity, and diminished survival outcomes [4, 5, 8]. These disparities are exacerbated by age‐associated comorbidities, reduced tolerance to intensive chemotherapy, and sociodemographic barriers to optimal care, underscoring the unmet need for age‐adapted prognostic frameworks capable of integrating geriatric‐specific vulnerabilities into risk stratification. This study establishes the first dual survival endpoint (OS and CSS) prognostic nomogram specifically designed for middle‐aged and elderly BL patients, leveraging population‐level data from the SEER registry. By integrating demographic (age, race), clinical (Ann Arbor stage), and treatment modality utilization (chemotherapy) predictors, the model identifies distinct risk stratification within this understudied cohort. Demonstrating robust discriminative capacity (AUC 0.71–0.81), exceptional calibration (slope ≈ 1.0), and significant net clinical benefit, this tool enables precise risk stratification and survival prediction in geriatric BL populations.

Age at diagnosis represents a pivotal prognostic determinant in adult BL. Retrospective analyses consistently demonstrate marked survival disparities between patients aged < 40 years and those ≥ 40 years, though outcomes remain comparable between subgroups aged 40–59 and ≥ 60 years [4, 12, 13]. This divergence likely reflects age‐related disparities in disease presentation, comorbidities, diminished baseline performance status, and reduced tolerance to intensive chemotherapy regimens. Additionally, geriatric BL exhibits higher frequencies of complex cytogenetic profiles, potentially altering tumor biology and therapeutic responsiveness [14]. Notably, 20% of elderly patients in our cohort received no chemotherapy—a trend aligning with population‐level data showing a 5.3% annual decline in systemic therapy utilization per year of advancing age [5]. This underutilization of systemic therapy, reflecting clinical risk–benefit assessments and patient preferences, underscores unmet needs in geriatric BL management. Our continuous‐variable analysis confirmed a monotonic mortality risk escalation with aging (HR = 1.03/year), reinforcing age as an independent survival predictor.

The Ann Arbor system, initially devised for Hodgkin lymphoma, remains widely applied to NHL including BL [7]. Consistent with BL's aggressive biology, disease stage demonstrated predominant prognostic significance. Advanced‐stage (III/IV) disease prevalence reached 66% in our middle‐aged/elderly cohort, aligning with prior adult BL reports [4, 15]. The persistent independent prognostic value of staging in multivariate analysis suggests exacerbated stage‐dependent survival disparities in older versus younger BL patients. This may reflect two age‐related mechanisms: (1) frequent use of attenuated chemotherapy regimens in elderly patients to mitigate toxicity, potentially undermining disease control in advanced stages; and (2) comorbid‐driven depletion of organ reserve capacity, hastening multiorgan dysfunction in late‐stage disease.

Multivariable analysis confirmed racial disparities as an independent predictor of OS in BL, consistent with observations in aggressive lymphomas such as diffuse large B‐cell lymphoma, where Black patients exhibit inferior survival outcomes versus White counterparts independent of disease stage [15, 16]. We found that American Indian race within the “Other” category had a HR of 2.35 (95% CI: 1.05–5.29) taking white ethnicity as a reference (Table S3). Given the small sample size of fewer than 20 cases, larger studies are needed to confirm this prognostic value. The observed interethnic survival differences likely reflect a multifactorial etiology encompassing genetic predisposition, socioeconomic inequities in healthcare access, and treatment adherence disparities [17, 18].

Chemotherapy remains the cornerstone of BL management, with treatment protocols evolving from the shared pathobiology between BL and B‐cell acute lymphoblastic leukemia [19, 20]. While pediatric‐inspired dose‐intensive regimens achieve efficacy in adults, treatment‐related toxicities necessitate frequent dose attenuation in older adults. Retrospective analyses demonstrate retained clinical benefit in older patients (25% aged > 60 years), with 65%–80% 2‐year event‐free survival (EFS) rates [21, 22, 23, 24]. The risk‐adapted DA‐EPOCH‐R regimen—utilizing prolonged low‐dose drug exposure to minimize peak toxicity while preserving efficacy—has emerged as a promising strategy. Prospective multicenter studies report 85% 4‐year EFS with DA‐EPOCH‐R, including efficacy in geriatric and HIV‐associated BL subgroups [25, 26]. Although intensive chemotherapy is the only potentially curative option for BL, our nomogram incorporates chemotherapy receipt as a proxy for real‐world treatment accessibility and patient fitness. This variable captures systemic disparities rather than biological susceptibility, explaining its prognostic weight. Its inclusion aims to identify undertreated cohorts needing supportive care escalation.

Radiotherapy (RT) now holds a marginal role in BL management, reserved for risk‐adapted contexts: consolidative local therapy for bulky/residual disease, palliative symptom control, or prophylactic sanctuary site irradiation [27, 28, 29, 30, 31]. In the context of our model, “RT” likely serves less as a direct causative factor for survival and more as a surrogate marker for distinct patient subgroups and treatment response. Patients who received RT were typically those with less disseminated disease (e.g., solitary testicular or orbital involvement) or those who had responded sufficiently to systemic therapy to be candidates for local consolidation. This identifies a clinically relevant subgroup with a better overall prognosis, which is particularly valuable for stratifying elderly patients. While our analysis associated RT with CSS improvement (HR = 0.74, *p* = 0.013), it lacked OS benefit—a discrepancy likely reflecting selection bias (RT recipients' higher baseline mortality risk partially offset by locoregional control) and competing mortality risks. In elderly patients, RT‐associated toxicities (neurotoxicity, myelosuppression) may elevate non‐lymphoma mortality, negating survival gains [32]. Though select subgroups (e.g., CNS/testicular involvement) may derive benefit, the SEER database's lack of radiation field specification precludes subgroup analysis, attenuating population‐level effect estimates.

While this study provides clinically valuable prognostic insights for middle‐aged and elderly BL patients, there are certain limitations. Firstly, the study is based on data from the SEER database, which may not capture all detailed clinical characteristics that could influence BL prognosis, such as lactate dehydrogenase levels, performance status, CNS involvement, and HIV infection. Secondly, information regarding treatment is limited. Detailed information on many variables such as chemotherapy regimen and intensity is missing. The SEER database lacks detailed information on radiotherapy intent (curative vs. palliative), start dates, dosage, and field targets. Therefore, a granular analysis to fully disentangle selection bias (e.g., through time‐dependent modeling) was not feasible. Our “RT yes/no” variable should be interpreted as a broad indicator of treatment patterns rather than a specific therapeutic recommendation. Third, the study population is limited to the United States, and the results may not be generalizable to other populations with different demographic characteristics and healthcare systems. External validation in Asian populations is ongoing.

## 5 Conclusion

This study establishes and validates a prognostic nomogram addressing critical gaps in risk‐stratified management of middle‐aged and elderly BL patients. While demonstrating robust discrimination (AUC 0.71–0.81) and calibration, external multicenter validation remains essential to confirm generalizability. Future iterations should incorporate genomic and radiomic biomarkers through multiomics integration, advancing precision therapeutics to optimize survival outcomes in aging populations.

## Author Contributions

**Xia Cao:** conceptualization, formal analysis, writing – original draft. **Duanzong Zhang:** conceptualization, writing – original draft. **Jichang Gong:** methodology, investigation. **Xin Yang:** formal analysis, data curation. **Jiqiong He:** methodology. **Yaqiong Li:** conceptualization, validation. **Hongjiang Pu:** conceptualization, validation, supervision. All authors reviewed and approved the final manuscript.

## Ethics Statement

The SEER database, a publicly available cancer surveillance database maintained by the United States National Cancer Institute, provides anonymized tumor‐related data to researchers at no cost. All patient data included in the SEER database was ethically approved by the National Cancer Institute in accordance with institutional protocols.

## Conflicts of Interest

The authors declare no conflicts of interest.

## Supporting information


**Table S1:** Univariate COX regression analysis of OS predictors in training set.
**Table S2:** Univariate COX regression analysis of CSS predictors in training set.
**Table S3:** Multivariate COX regression analysis of OS predictors in training set based on racial variable four‐class classification.
**Table S4:** Results of hypothesis testing for association between Ann Arbor stage and tumor number.

## Data Availability

The data that support the findings of this study are available in the SEER database.
