# Supplementary material for: Survival Prediction in Middle‐Aged and Elderly Patients With Burkitt Lymphoma: A Comprehensive Nomogram Approach Based on SEER Data
Source: Cancer Med. 2025 Nov 10;14(21):e71334. doi: 10.1002/cam4.71334 (PMC12599551; doi:10.1002/cam4.71334)
Supplement: Supplementary file 1 — Table S1: Univariate COX regression analysis of OS predictors in training set. Table S2: Univariate COX regression analysis of CSS predictors in training set. Table S3: Multivariate COX regression analysis of OS predictors in training set based on racial variable four‐class classification. Table S4: Results of hypothesis testing for association between Ann Arbor stage and tumor number. [file CAM4-14-e71334-s001.docx]

**Table S1.** Univariate COX regression analysis of OS predictors in training set.

| **Characteristic** | **HR**^1^ | **95% CI**^1^ | **p-value** |
| --- | --- | --- | --- |
| Sex |  |  |  |
| Male | — | — |  |
| Female | 1.06 | 0.95, 1.19 | 0.275 |
| Age | 1.04 | 1.03, 1.04 | <0.001 |
| Race |  |  |  |
| Black | — | — |  |
| Other | 0.91 | 0.71, 1.17 | 0.447 |
| White | 0.78 | 0.64, 0.96 | 0.016 |
| Marital status |  |  |  |
| Divorced | — | — |  |
| Married | 1.19 | 0.97, 1.47 | 0.102 |
| Separated | 1.09 | 0.63, 1.87 | 0.760 |
| Unmarried | 1.19 | 0.95, 1.49 | 0.135 |
| Widowed | 2.12 | 1.66, 2.71 | <0.001 |
| Household income |  |  |  |
| 50K-75K | — | — |  |
| Greater than 75k | 0.95 | 0.85, 1.06 | 0.386 |
| Less than 50,000 | 1.34 | 1.06, 1.70 | 0.014 |
| Ann Arbor Stage |  |  |  |
| I | — | — |  |
| II | 1.08 | 0.87, 1.35 | 0.473 |
| III | 1.52 | 1.21, 1.91 | <0.001 |
| IV | 1.70 | 1.44, 2.01 | <0.001 |
| Radiation |  |  |  |
| No | — | — |  |
| Yes | 0.92 | 0.76, 1.11 | 0.392 |
| Chemotherapy |  |  |  |
| No | — | — |  |
| Yes | 0.24 | 0.21, 0.27 | <0.001 |
| Diagnosis to treatment time |  |  |  |
| ≤1 month | — | — |  |
| ＞1 month | 0.89 | 0.74, 1.08 | 0.254 |
| Primary Site |  |  |  |
| lymphatic node | — | — |  |
| Extra-lymph node | 0.92 | 0.82, 1.03 | 0.133 |
| Laterality |  |  |  |
| Unilateral | — | — |  |
| Bilateral | 0.70 | 0.58, 0.86 | <0.001 |
| First malignant primary |  |  |  |
| No | — | — |  |
| Yes | 0.67 | 0.59, 0.77 | <0.001 |
| number of tumor masses |  |  |  |
| ≥2 | — | — |  |
| 1 | 0.94 | 0.91, 1.18 | 0.488 |

^1^HR = Hazard Ratio, CI = Confidence Interval

**Table S2.** Univariate COX regression analysis of CSS predictors in training set.

| **Characteristic** | **HR**^1^ | **95% CI**^1^ | **p-value** |
| --- | --- | --- | --- |
| Age | 1.01 | 1.01, 1.02 | <0.001 |
| Sex |  |  |  |
| Female | — | — |  |
| Male | 1.04 | 0.91, 1.18 | 0.575 |
| Race |  |  |  |
| Black | — | — |  |
| Other | 0.97 | 0.82, 1.14 | 0.685 |
| White | 1.02 | 0.81, 1.29 | 0.880 |
| Marital status |  |  |  |
| Divorced | — | — |  |
| Married | 1.14 | 0.91, 1.43 | 0.259 |
| Separated | 1.35 | 0.78, 2.34 | 0.283 |
| Unmarried | 1.16 | 0.90, 1.48 | 0.249 |
| Widowed | 1.22 | 0.93, 1.59 | 0.152 |
| Household income |  |  |  |
| 50K-75K | — | — |  |
| Greater than 75k | 0.92 | 0.82, 1.05 | 0.206 |
| Less than 50,000 | 0.97 | 0.74, 1.26 | 0.808 |
| Ann Arbor Stage |  |  |  |
| I | — | — |  |
| II | 1.18 | 0.90, 1.54 | 0.227 |
| III | 1.18 | 0.90, 1.54 | 0.225 |
| IV | 1.54 | 1.26, 1.89 | <0.001 |
| Radiation |  |  |  |
| No | — | — |  |
| Yes | 0.69 | 0.55, 0.86 | <0.001 |
| Chemotherapy |  |  |  |
| No | — | — |  |
| Yes | 0.34 | 0.30, 0.39 | <0.001 |
| Diagnosis to treatment time |  |  |  |
| ＞1 month | — | — |  |
| ≤1 month | 1.13 | 0.91, 1.41 | 0.263 |
| Primary Site |  |  |  |
| Extra-lymph node | — | — |  |
| lymphatic node | 1.02 | 0.90, 1.16 | 0.734 |
| Laterality |  |  |  |
| Unilateral | — | — |  |
| Bilateral | 0.64 | 0.49, 0.82 | <0.001 |
| First malignant primary |  |  |  |
| No | — | — |  |
| Yes | 0.92 | 0.79, 1.08 | 0.312 |
| number of tumor masses |  |  |  |
| 1 | — | — |  |
| ≥2 | 1.42 | 1.24, 1.64 | <0.001 |

^1^HR = Hazard Ratio, CI = Confidence Interval

**Table S3.** Multivariate COX regression analysis of OS predictors in training set based on racial variable four-class classification.

| **Characteristic** | **HR**^1^ | **95% CI**^1^ | **p-value** |
| --- | --- | --- | --- |
| Age | 1.03 | 1.03, 1.04 | <0.001 |
| Race |  |  |  |
| White | — | — |  |
| Black | 1.38 | 1.11, 1.72 | 0.003 |
| American Indian | 2.35^a^ | 1.05, 5.29 | 0.038 |
| Asian or Pacific Islander | 0.96 | 0.78, 1.18 | 0.674 |
| Marital status |  |  |  |
| Divorced | — | — |  |
| Married | 1.09 | 0.86, 1.39 | 0.482 |
| Separated | 1.52 | 0.86, 2.70 | 0.150 |
| Unmarried | 1.22 | 0.94, 1.59 | 0.141 |
| Widowed | 0.99 | 0.74, 1.34 | 0.959 |
| Ann Arbor Stage |  |  |  |
| I | — | — |  |
| II | 1.30 | 1.04, 1.63 | 0.021 |
| III | 1.87 | 1.47, 2.38 | <0.001 |
| IV | 2.27 | 1.90, 2.72 | <0.001 |
| Chemotherapy |  |  |  |
| No | — | — |  |
| Yes | 0.26 | 0.22, 0.30 | <0.001 |
| Laterality |  |  |  |
| Unilateral | — | — |  |
| Bilateral | 0.91 | 0.71, 1.15 | 0.422 |
| First malignant primary |  |  |  |
| No | — | — |  |
| Yes | 0.97 | 0.82, 1.15 | 0.710 |

^1^HR = Hazard Ratio, CI = Confidence Interval

^a^HR of 2.35 for the American Indian race should be interpreted with caution due to the small sample size

**Table S4.** Results of Hypothesis Testing for Association Between Ann Arbor Stage and Tumor Number

| Testing Method | Statistic | P-value | Degrees of Freedom |
| --- | --- | --- | --- |
| Chi-Square Test | 6.27898108 | 0.09879856 | 3 |
| Cramer's V | 0.05349661 |  |  |
